# Recurrence of Pigmented Epithelioid Angiomyolipoma of the Kidney With Xp11 Translocation: A Case Report

**DOI:** 10.7759/cureus.47305

**Published:** 2023-10-19

**Authors:** Mahmoud D Srour, Andrew Harris

**Affiliations:** 1 Urology, Great Western Hospital NHS Foundation Trust, Swindon, GBR

**Keywords:** xp11 translocation pecoma, laproscopic nephrectomy, local excision, adequate follow-up, renal malignant epithelioid angiomyolipoma

## Abstract

This case report pertains to a 70-year-old male patient with a medical history marked by atrial fibrillation, ankylosing spondylitis, and Crohn's disease. Eight years prior, the patient underwent a left radical nephrectomy due to the presence of a pigmented epithelioid angiomyolipoma (PEComa) in the kidney. Notably, pathological examination revealed an unusual subtype of PEComa characterized by Xp11 gene translocation, indicating a more aggressive clinical profile. Following a five-year observation period without recurrence, the patient was discharged.

However, eight years after initial treatment, he presented with vague symptoms of left loin discomfort and fullness, which had persisted for several weeks. Subsequent evaluation via computed tomography (CT) scanning showed a small lesion at the site of the renal bed. Surgical resection confirmed the return of the identical tumour. Key clinical points elucidated by this case include the varied behaviour of PEComas, the essential need for prolonged surveillance, and a recognition that recurrences can transpire even after extended disease-free intervals. Prior studies suggest recurrence rates of up to 31.8% for this specific PEComa subtype, emphasising the requirement for prolonged follow-up protocols.

## Introduction

Perivascular epithelioid cell tumours (PEComas) are a group of rare mesenchymal neoplasms composed of perivascular epithelioid cells exhibiting both melanocytic and muscular differentiation. The majority of PEComas show genetic alterations in TSC2 (the result of a loss of heterozygosity in the TSC2 gene) and, less commonly, TSC1. A small subset of PEComas harbour TFE3 (Xp11) gene fusions, which show unique characteristics and are considered to represent an unusual variant of PEComa [1]. Perivascular epithelioid cell tumours of the urinary system and male genital organs are extremely rare mesenchymal neoplasms that have been described mainly in the kidney [2].

We present a case of recurrence of Xp11 translocation PEComa occurring in the kidney harbouring TFE3 rearrangement in a 70-year-old male, which is an exceedingly rare variant of the rare PEComa [3-4].

## Case presentation

**Figure 1:**
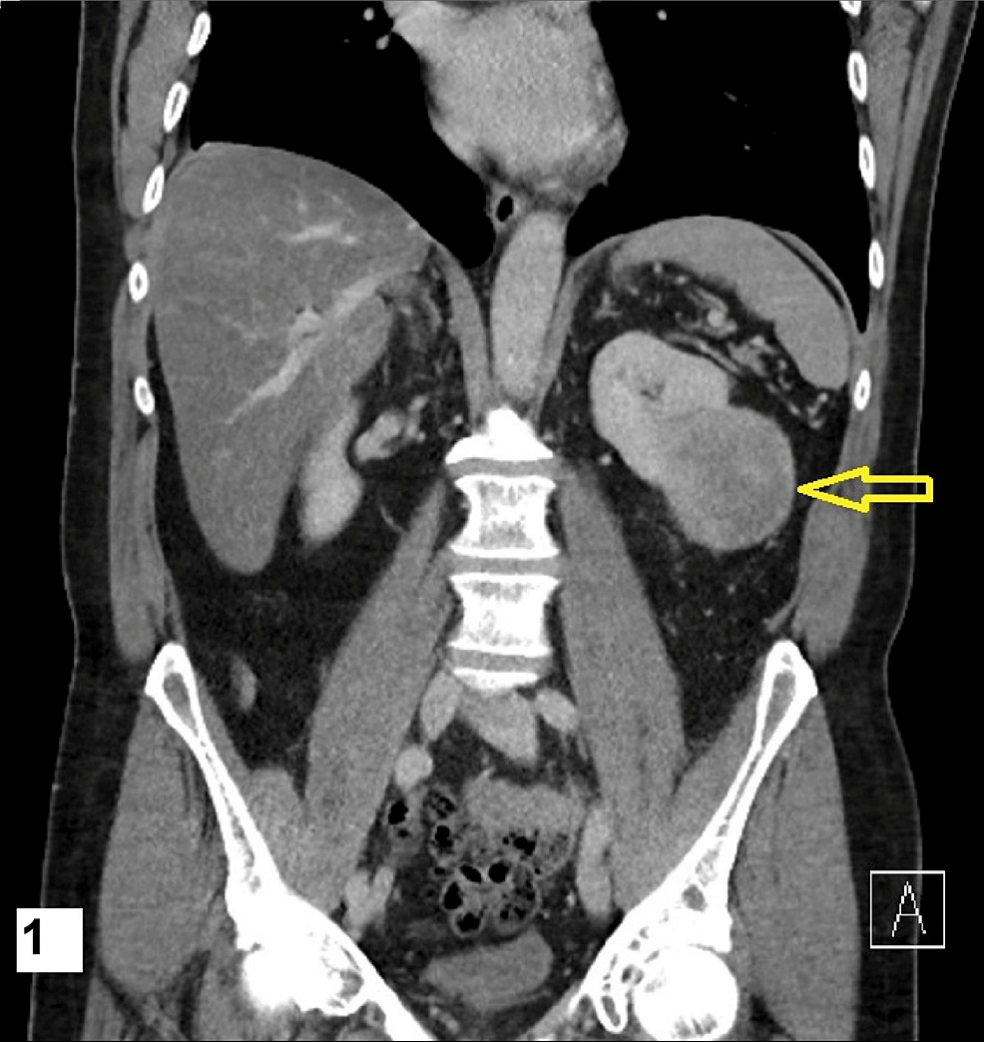
Coronal image of the CT scan showing the original tumour The yellow arrow points to the left kidney tumour.

The patient is a 70-year-old male with a history of atrial fibrillation, ankylosing spondylitis, and Crohn's disease. Eight years prior, he underwent a left radical nephrectomy for a solitary, non-metastatic 67mm tumour in its maximum dimension with focal necrosis and a dark brown cut surface (Figure 1).

**Figure 2:**
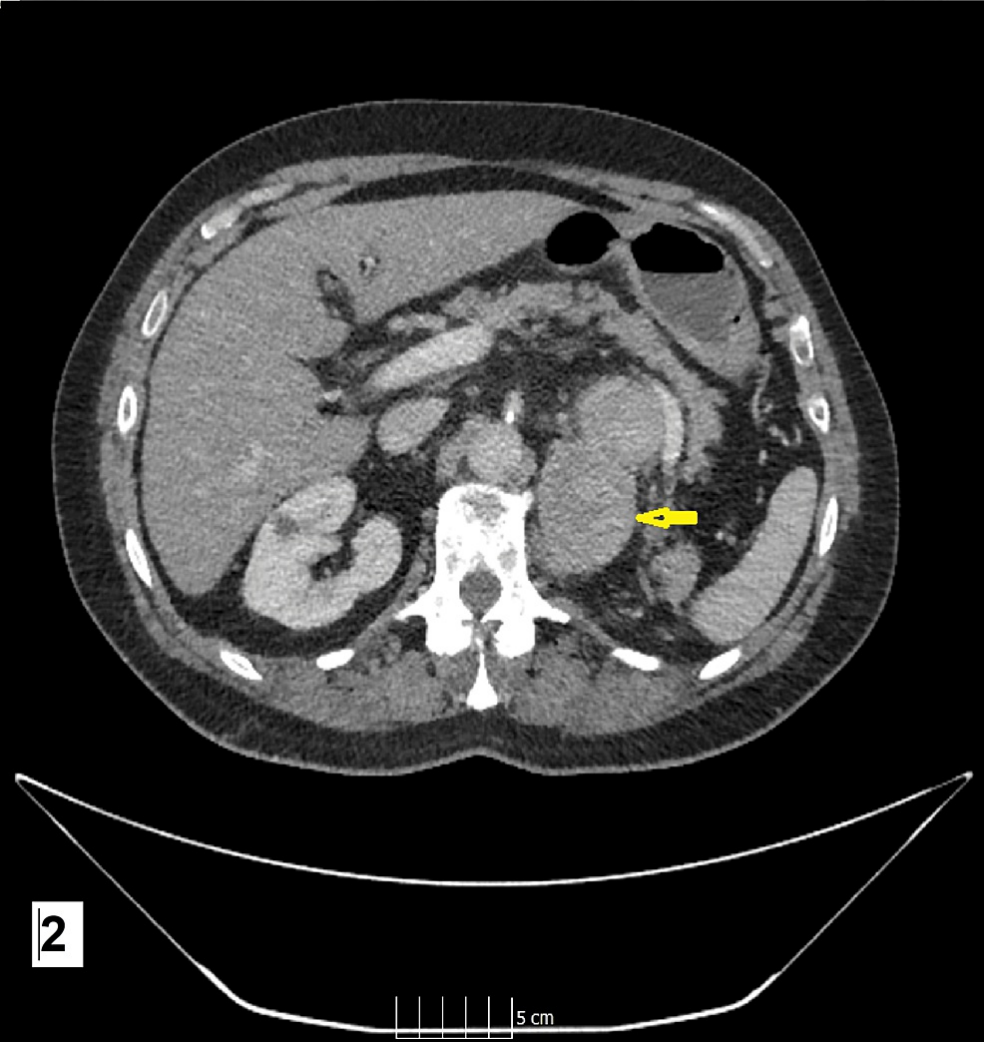
Axial image of the CT scan showing the tumour recurrence site The yellow arrow points to the left nephrectomy bed recurrence.

**Figure 3:**
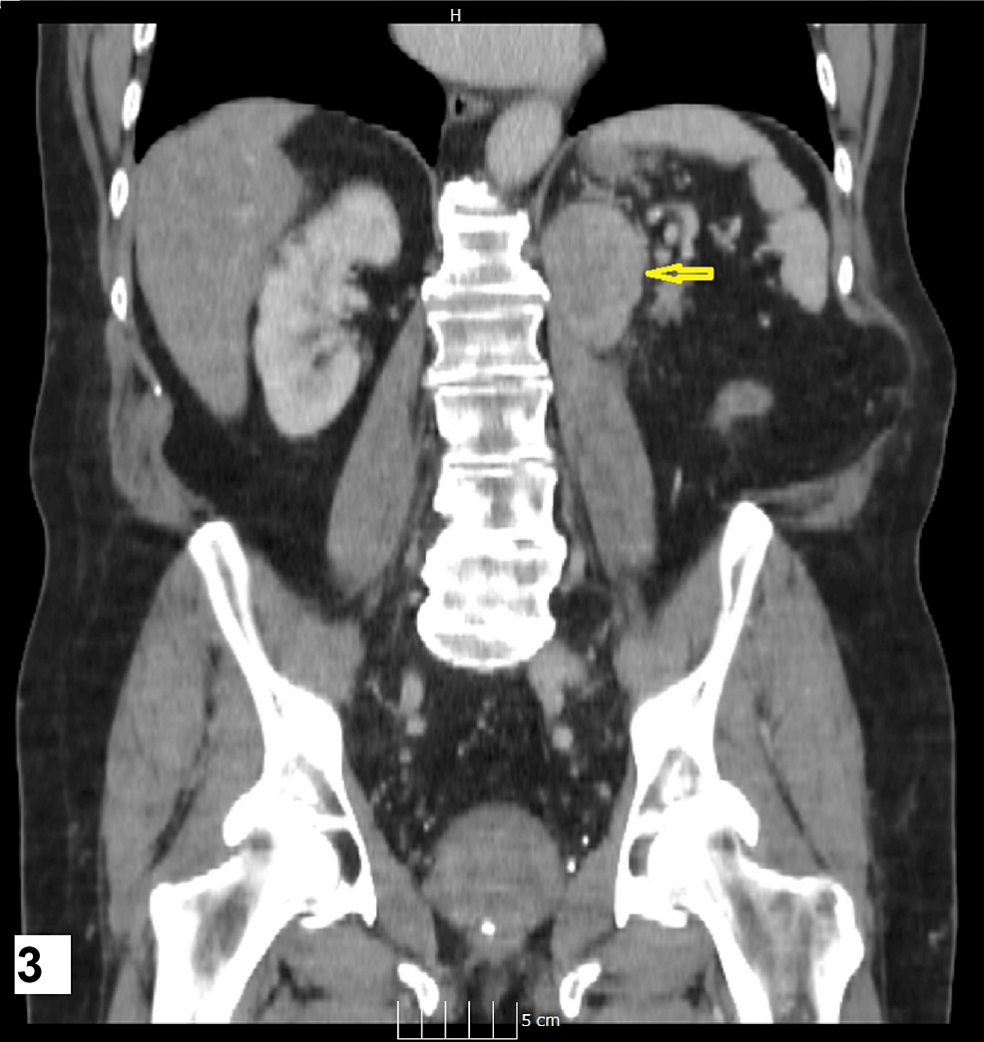
Coronal image of the CT scan showing the tumour recurrence site The yellow arrow points to the left nephrectomy bed recurrence.

The tumour was well-circumscribed. It had a rather solid architecture composed of sheets of plump epithelioid cells. Under the microscope, the tumour cells had a granular cytoplasm, which was relatively abundant. The nuclei were centrally placed and showed considerable pleomorphism. Nucleoli were prominent, but mitotic figures were infrequent. There was iron pigment, but the black pigment had the colour and staining properties of melanin. There were areas of haemorrhage and necrosis. The Azzopardi phenomenon of haematoxophilic presumed DNA fragments surrounding blood vessels was seen. There was lymphovascular infiltration present. Within the tumour, there were some thick-walled blood vessels with intense eosinophilia. Tumour cells stained intensely for HMB-45 but not melan-A. They were cytokeratin (CK)-negative. There was B-cell lymphoma 2 (Bcl-2) staining and occasional Ki67-positive cells. Inhibin, chromogranin A, CD56, CD31, synaptophysin, CD10, CD15, CK7, and epithelial membrane antigen (EMA) were all negative. It was also stained for smooth muscle markers and found to be negative; CD68 was negative, but most importantly, TFE3 showed strong positive staining for nuclei. These were the several features of a pigmented epithelioid angiomyolipoma with an Xp11 chromosome translocation involving the TFE3 gene. The case was discussed with the multidisciplinary team, and due to the rarity, the little information known about this variant, and the fact that the lesion was well-circumscribed and exophytic in nature, no further treatments were offered. The patient remained under regular surveillance with yearly interval CT scans for five years, during which he was asymptomatic and showed no signs of disease recurrence. He was discharged after that. However, the patient recently complained of left-loin fullness and discomfort. A CT scan was performed, revealing a lesion in the renal bed suggestive of recurrence (Figures 2-3).

Subsequent robotic surgical resection of the lesion was carried out without significant complications. Histopathological examination confirmed the recurrence of the same disease. It is an exceedingly rare histological variant tumour, which makes effective systemic therapies limited [5]. Furthermore, the patient has a history of Crohn’s, rendering radiotherapy an unfavourable treatment option.

## Discussion

This case also underscores the aggressive nature of Xp11 translocation PEComas. With a propensity for metastasis in more than 30% of cases, it carries a significant risk of disease progression, making an early and accurate diagnosis all the more imperative. The strong nuclear staining for TFE3, a key diagnostic marker for this variant, aids in its identification, guiding clinical decision-making.

The recommended follow-up period for patients with PEComa is typically five years. However, this case brings to light an important caveat: recurrence can occur beyond this time frame. This finding aligns with other reports in the literature, where recurrences have been observed as late as seven to nine years after initial treatment [6]. Therefore, this case underscores the need for an extended surveillance period, extending follow-up beyond five years.

The optimal duration of follow-up for PEComa patients remains a topic of debate, as the rarity of this tumour makes it challenging to establish definitive guidelines. Nonetheless, this case strongly suggests that for this specific variant with Xp11 translocation, at least a 10-year follow-up period is advisable. This prolonged follow-up duration is essential for early detection of recurrences, thereby facilitating timely intervention.

Furthermore, the patient's history of Crohn's disease presents an additional layer of complexity. Given the unfavourable nature of radiotherapy for Crohn's patients, alternative treatment options should be considered. The limitations of available systemic therapies for this exceedingly rare histological variant further emphasise the importance of surveillance and early detection as a primary means of managing the disease [7,8].

## Conclusions

In conclusion, this case report illuminates the nature of an exceedingly rare variant of PEComa with Xp11 translocation. The distinctive histological and molecular features of this tumour necessitate precise diagnosis and specialised clinical management. Furthermore, the aggressive clinical profile and a noteworthy risk of metastasis underline the urgency of vigilant monitoring and early intervention. The essential message from this case is the imperative need for extended surveillance, extending beyond the conventional five-year follow-up period. Patients with this rare PEComa variant should be offered tailored care, considering the unique challenges it presents, particularly when coexisting conditions, like Crohn's disease, are involved. As a complex and rare entity, the need for further research is important to develop more concrete guidelines for the management of such cases and enhance our understanding of this exceptionally rare tumour subtype.

## References

[1] Argani P, Aulmann S, Illei PB (2010). A distinctive subset of PEComas harbors TFE3 gene fusions. Am J Surg Pathol.

[2] Chang H, Jung W, Kang Y, Jung WY (2012). Pigmented perivascular epithelioid cell tumor (PEComa) of the kidney: a case report and review of the literature. Korean J Pathol.

[3] Saoud R, Kristof TW, Judge C, Chumbalkar V, Antic T, Eggener S, Modi P (2022). Clinical and pathological features of renal epithelioid angiomyolipoma (PEComa): a single institution series. Urol Oncol.

[4] Wang XT, Fang R, Zhang RS (2020). Malignant melanotic Xp11 neoplasms exhibit a clinicopathologic spectrum and gene expression profiling akin to alveolar soft part sarcoma: a proposal for reclassification. J Pathol.

[5] Zhang H, Wang S, Meng L (2023). Primary Xp11 translocation PEComa of the testis with SFPQ⁃TFE3 rearrangement: a case report and review of the literature. Diagn Pathol.

[6] Martignoni G, Pea M, Reghellin D, Zamboni G, Bonetti F (2008). PEComas: the past, the present and the future. Virchows Arch.

[7] Brimo F, Robinson B, Guo C, Zhou M, Latour M, Epstein JI (2010). Renal epithelioid angiomyolipoma with atypia: a series of 40 cases with emphasis on clinicopathologic prognostic indicators of malignancy. Am J Surg Pathol.

[8] Zhang J, Wang WJ, Chen LH (2023). Primary renal malignant epithelioid angiomyolipoma with distant metastasis: a case report and literature review. Front Oncol.

